# Comparison of efficiency of PFA catheter designs by computer modeling

**DOI:** 10.1111/jce.16459

**Published:** 2024-10-08

**Authors:** Andres Belalcazar, E. Kevin Heist

**Affiliations:** ^1^ Biophysicist Minneapolis Minnesota USA; ^2^ Cardiac Arrhythmia Service Massachusetts General Hospital Boston Massachusetts USA

**Keywords:** atrial Fibrillation, electroporation, pulmonary vein isolation, pulsed field ablation

## Abstract

**Introduction:**

Various catheter designs are appearing for Pulsed Field Ablation (PFA). It is unclear if they differ in terms of safety and efficiency. PFA studies have reported hemolysis, kidney injury, high troponin, among other side effects.

**Methods:**

Using a CT‐derived computer model, we compared catheter designs using two metrics: (1) efficiency: power delivered to an atrial wall target, expressed as a percent of total generator power; and (2) safety: electric current to achieve 90% transmurality (since more energy causes more collateral effects), as well as the corresponding electrode current density (ECD), a heat and bubble metric. The following catheter designs were compared: penta‐spline basket, Nitinol spheres (focal 9 mm and large 1‐shot), circular, balloon, and flex‐circuit. Target was a 6 × 47 mm circumferential segment of atrial wall at LPV antrum. Transmurality was defined as percent of target having >600 volts per centimeter (V/cm) electric field needed for electroporation.

**Results:**

Efficiency was 0.9, 1.4, 2.7, 5.9, 10, and 12% for the large 1‐shot and 9 mm Nitinol spheres, penta‐spline, circular, flex spline, and balloon catheters, respectively. Regarding safety, currents for 90% transmurality were 70, 39,36,12.5, 5.3, and 4 Amps for the same respective catheters, with less being safer. ECD was 124, 25, 74, 83, 41, and 31 A/cm^2^, respectively.

**Conclusion:**

Computer models demonstrated a remarkable range in efficiency among catheters studied. Those having less atrial blood exposure had the highest efficiencies, with factors of up to 13X more efficiency compared to exposed ones. Higher efficiency designs have less collateral current and are safer. Confirmatory in‐vivo studies are required.

## Graphical abstract

Central Illustration. PFA catheter designs have variable targeting and efficiency: two examples.

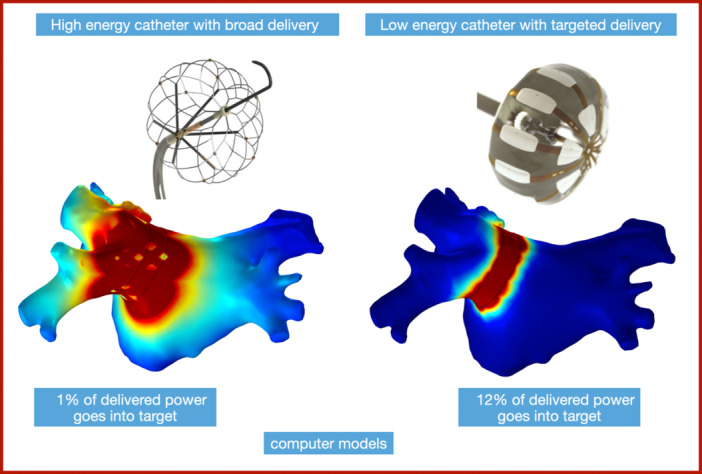

## 1 INTRODUCTION

**Figure 1:**
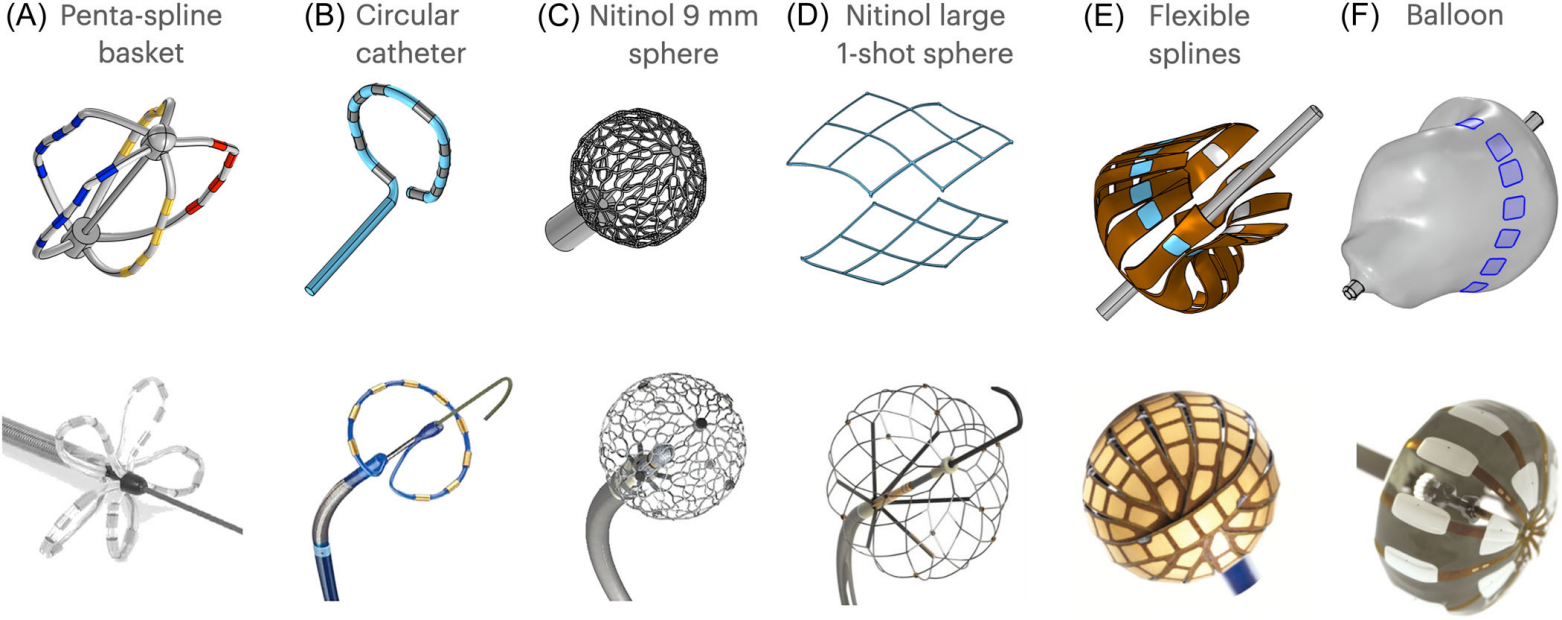
Catheter designs being compared. Some of these are commercially available by country, and some only for RF ablation. The top figures illustrate how our computer models rendered them, using information from patents and other publications. The Nitinol Large 1‐shot sphere consists of six “diamond panels” that are individually energized, two are shown here. Catheters are rendered deformed to contact the anatomy ideally, see Figure 2. *(Photo A‐C, from diBiase* et al ^2^ *with permission, copyright Elsevier 2021. Photo D from Koruth* et al ^7^ *Open Access, Creative Commons BY‐NC license 2023. Photo E from Turagam* et al ^8^ *Open Access, Creative Commons BY‐NC license 2023. Photo F from Musikantow* et al ^9^ *with permission, copyright Elsevier 2022.)*.

A variety of catheter designs using high voltage pulsed field ablation (PFA) have appeared in the market and in pivotal studies for the treatment of atrial fibrillation (AF).^1^, ^2^, ^3^ It is unclear if they differ substantially in terms of dose, safety, and efficiency. Side effects have been reported such as kidney injury ^4^ and high troponin,^5^ among others. Some catheters have small area electrodes while others are larger. There is also variation in spacing between electrodes, as well as in manners of energizing them. Some operate in a monopolar manner (return patch on back), while others do so in bipolar fashion. While the task of comparing these designs appears to be obstructed by the secrecy and proprietary nature of the manufacturers' waveforms, we propose that it is possible to solidly compare the catheter design via computer modeling if only vectoring is known. We previously reported on circular catheters and balloons,^6^ but not Nitinol spheres, nor penta‐spline designs, which will be reported here, with respect to pulmonary vein isolation (PVI). Figure 1 shows the PFA catheters studied. Using the catheter's instructions for use (IFU), publications, photographs, video from live cases at cardiology conferences, and patents, we reproduced similar ones in our models, as shown in the corresponding renderings in Figure 1.

## 2 METHODS

### 2.1 Computer model

**Figure 2:**
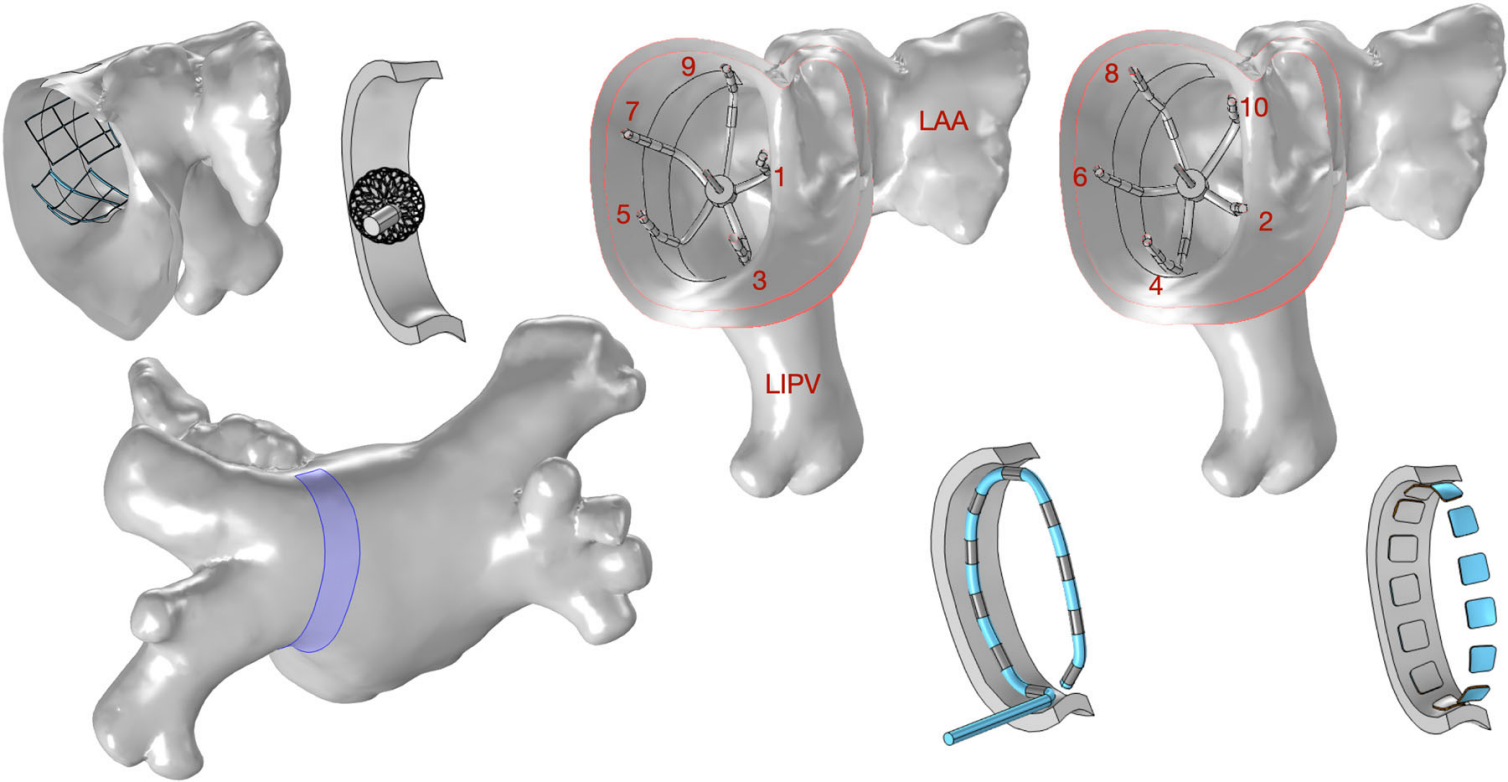
Target used in safety and efficiency assessment. The target is a 6 × 47 mm rectangular section of the posterior LPV antrum. All catheters compared were equally and ideally positioned ideally against it, conforming to the target anatomy. The large 1‐shot has three panels on target. The focal 9 mm sphere was applied seven times, every 6 mm, along the target's length. The penta‐spline includes two applications with an intervening rotation, with a total of six splines/vectors on the target. The circular catheter has three positive electrodes (or tripole vectors) on target. The flex splines/balloon catheters have four tripole vectors on target, with a rotation, for a total of 8. Only the energy applied posteriorly by the near‐target electrodes was accounted to compare the catheters, so that anterior energy deliveries did not count as wasted energy. This makes for fair comparisons versus the focal 9 mm sphere catheter, which is exclusively posterior.

A computer model built from thoracic CT images with contrast was used for comparisons. No ethical approvals were required for this study, as it uses imaging from an anonymized, publicly available radiology database. The details of its radiology source and modeling assumptions can be found in our earlier publication.^6^ To recap briefly, all organs near the target were included in detail, including atria and atrial walls (2.5 mm thickness), aortic walls, pulmonary arteries and veins, lungs, major airways, esophagus and coronary sinus, among others. A posterior wall of the left pulmonary vein (LPV) antrum was selected as the target. Figure 2 shows this target and how the catheters were applied to it. Comsol software (version 6.1) was used to numerically solve for electric fields and currents throughout the thorax, while the energy delivered was varied, with currents ranging from 2 to 70 Amp. Organs and tissues were segmented, converted to a finite element model, and assigned conductivity properties according to well‐known databases and publications.^6^ Tissues that experienced high voltages were modeled nonlinearly, meaning that their conductivity was not a constant, but depended on the electric field. It is well established that cellular membranes permeabilize at high electric fields causing a reduction in conductivity of tissue.^10^

### 2.2 Comparison without knowledge of waveforms

A preliminary objection to our work might be that comparisons of lesion creation versus energy cannot be studied if the precise waveform is unknown (manufacturers currently are unwilling to disclose their proprietary PFA waveforms). We submit that regardless of waveform, every catheter will deliver a percentage of its power into the target at any instant of time when energized, with the ideal in regard to ablation efficiency and safety being 100%. The actual percentage can be obtained via modeling.

**Figure 3:**
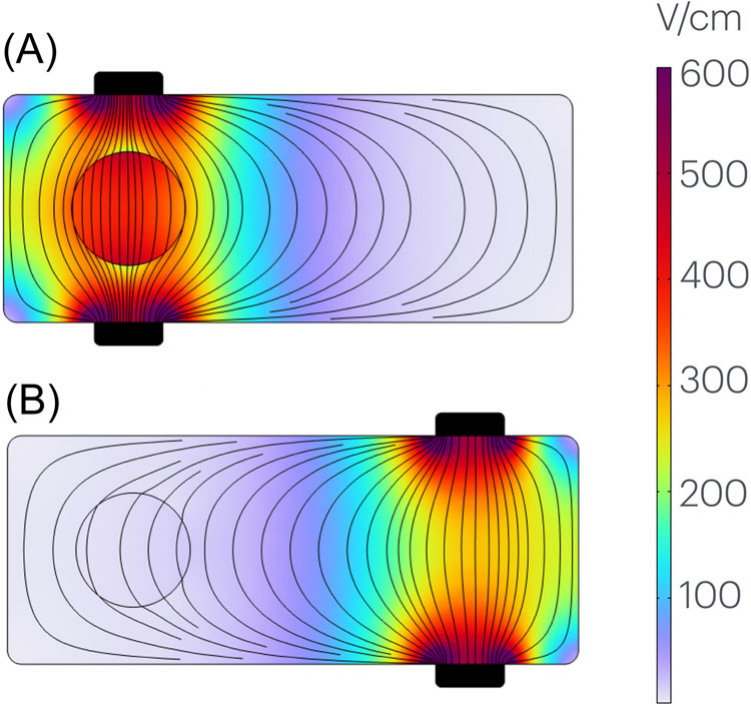
Why waveform knowledge is not needed to quantify PFA efficiency. In this simplified example, electric field lines are shown in a rectangular blood volume containing a circular myocardium target. In panel (A), the electrodes (in black) are better positioned to apply PFA fields to the circular target (circle). In contrast, the electrodes in panel (B) are farther from the target and thus provide weaker fields to it, but strong ones to nontarget tissue. It is evident that configuration A will be more efficient, because a greater percentage of the power from the electrodes is received at the target. The percent of power received at target in each case A and B can be quantified via modeling. In this way, no knowledge of waveform is needed for a quantitative comparison of power efficiency.

A first example in Figure 3 explains this central aspect of our methodology, which allows comparisons of catheter efficiency without waveform knowledge. The method is based on the fact that power is by definition an instantaneous quantity (i.e., the rate at which energy is being delivered). This basis liberates us from requiring pulse duration, number of pulses, or inter‐pulse delay. Similarly, we do not require the magnitude of power being delivered, since we instead focus on the fraction of the power injected into the body that is going to the target, regardless of total magnitude. Using those same instantaneous techniques, we can also evaluate what amount of instantaneous current is required to achieve 90% transmural electroporation in our target. No knowledge of waveform parameters is required for either of these two metrics (fraction of total power into target, current for 90% transmurality). Only vectoring is needed (see vectoring section later below).

Two further examples illustrate how our study manages without waveform parameters:

Example 2: two heat lamps are being compared, one has a back reflector behind its light bulb, the other has no reflector, allowing heat to radiate in all directions. If we were interested in forward heating (a target in front of the lamp) we could define efficiency as the ratio of output to input, with input power being that which goes into the lamp, and output being the thermal power received at the frontal target. The “waveform” from the lamp could vary in color, or use intermittent electricity, or a sinusoidal one, or in short, be any waveform, but we would still be able to measure a better efficiency ratio with the reflector lamp than with the bare bulb one. All that is needed is an instant of time with the lamps energized for us to quantify their cited ratio.

Example 3. Consider two focal catheters in a heart across the ventricular septum, one in the right ventricle, the other in the left ventricle. Imagine that PFA is applied between the two tips, bipolarly (no monopolar back patch involved). Now imagine that these catheter tips are pressed forcefully into the myocardium and come in complete electrical contact mid‐endocardially with each other, thereby creating a short circuit. It is self‐evident that no matter what waveform one uses, the efficiency of this arrangement is poor, if not zero. All current is wasted in the short circuit, none goes to tissue. Now, if we back off a little, and let the tips separate 1 mm for example, we will see that our efficiency is a bit improved relative to the short circuit case, but there is still much short‐circuit current with that small distance. Continue the separation to 5–10 mm, and we will see an improvement in lesion creation efficiency, because we are short circuiting less current and delivering more to the tissue. This efficiency behavior in essence has a mechanical and vectoring dependence. It will be observed regardless of waveform and can be studied quantitatively with computer modeling as in this study. Now, a comparison of two PFA systems as just described can be made, one with 1 mm separation, and another with 10 mm separation. Assuming the same unknown electroporating waveform is used in both cases, it is easy to see that the 10 mm system is inherently more efficient than the 1 mm one, an assertion that can be made and quantified without waveform knowledge. For these reasons, different catheter designs can be compared in regard to energy transmission to target tissue vs surrounding structures as a measure of catheter efficiency, in a waveform‐independent manner.

### 2.3 Clinical versus model waveforms, relationship to efficiency

The above is not to say that waveform is unimportant: it is an additional factor, ‐ combined with catheter design and efficiency‐, that will impact final clinical lesion dimensions, and risk for complications. But how do these clinical waveforms and lesions relate to those of this modeling study? We hypothesized that manufacturers have compensated waveforms when inherent inefficiencies may be found by our study. (Compensations can include adding applications, widening pulse widths, or increasing energy delivery per application). We therefore examined the consistency between our study's efficiency metrics and published energy durations in clinical studies: inefficient catheters are likely to have longer energy durations, we hypothesized.

Our study is not replicating actual lesions after manufacturer compensations and their true waveforms. Rather, we are comparing inherent catheter efficiencies that hypothetically depend on the different mechanical, geometrical, and vectoring approaches taken by manufacturers. We do so with an assumption of an equal waveform used across all catheters, a waveform that produces an electroporation lesion with 600 V/cm (see Section 2.6).

### 2.4 Catheter and vectoring assumptions

The dimensions and vectoring (electrodes and polarity used to deliver energy) of the circular catheter, flexible splines, and balloon catheters were described in detail in our previous publication.^6^ Catheter design, geometry, and electrode spacing were modeled as accurately as possible based on available patents and publications. To reiterate briefly, the circular catheter was a decapolar one with 3 mm long, 1.6 mm diameter ring electrodes, spaced at 3.7 mm. It was energized in an interlaced bipolar manner, with adjacent, alternating positive and negative electrodes. The flexible spline and balloon catheters used 3.6 x 3.6 mm electrodes, spaced every 6 mm, with the splines being 4 mm wide. In this report, we energized them using the best performing wide‐interlaced method we described in our previous publication. That is, two sequential energy deliveries, each with positive and negative alternating electrodes, with inactive electrodes between them. The reader is referred to our earlier publication ^6^ for vectoring diagrams and more details.

The penta‐spline catheter was modeled as an elongated basket from public photographs with each spline having four ring electrodes, each 1.3 mm in diameter, 3 mm in length, with interelectrode spacing of 3, 3 and 4 mm. All electrodes in a spline were connected together. We initially modeled the energy delivery vector as a rotating tripole per one of the manufacturer's patents,^11^ with one spline being an anode, and the diametrically opposed pair of splines as cathodes. But we later observed a live case from the Prague Rhythm 2022 conference ^12^ in which adjacent splines were positioned too close to each other, causing the generator to abort and give an alarm, a problem that was solved by separating the splines. This indicates that the vectoring cannot be tripolar, and is instead bipolar, from one spline to the adjacent spline. An educational video published by the manufacturer suggested the same.^13^ Nevertheless, we modeled both vectoring schemes and though they had similar results, present here the best performer, the bipole. Based on the electrogram traces of the live case showing five deliveries per application, the bipole was assumed to rotate five times per application.

For the focal 9 mm sphere, the Nitinol wire struts had a thickness of 0.127 mm, aiming to follow published photographs and patents.^14^, ^15^, ^16^ PFA energy is delivered in a “monopolar” fashion, from the sphere to a patch on the back of the patient. To model the larger 1‐shot sphere we followed a descriptive patent and a publication.^7^, ^17^ The sphere consists of six Nitinol panels that have “diamonds” in them (Figure 1). The panels are joined with insulators according to the patent, forming a sphere that is about 20–40 mm in diameter, depending on anatomical deployment. For its vectoring, we initially were led by an HRS PFA Webinar presentation ^18^ which showed the device operating in saline, with significant arcing and bubbles at the panel joints. This implied a bipolar, panel‐to‐adjacent‐panel vectoring scheme. In contrast, preclinical study publications of the device described a monopolar arrangement, with the six panels “independently and sequentially energized.”^7^ Given the uncertainty, we modeled both methods and present here the best‐performing method: the monopolar, sequential energization of each individual panel to a back patch.

### 2.5 Placement and electrode contact

**Figure 4:**
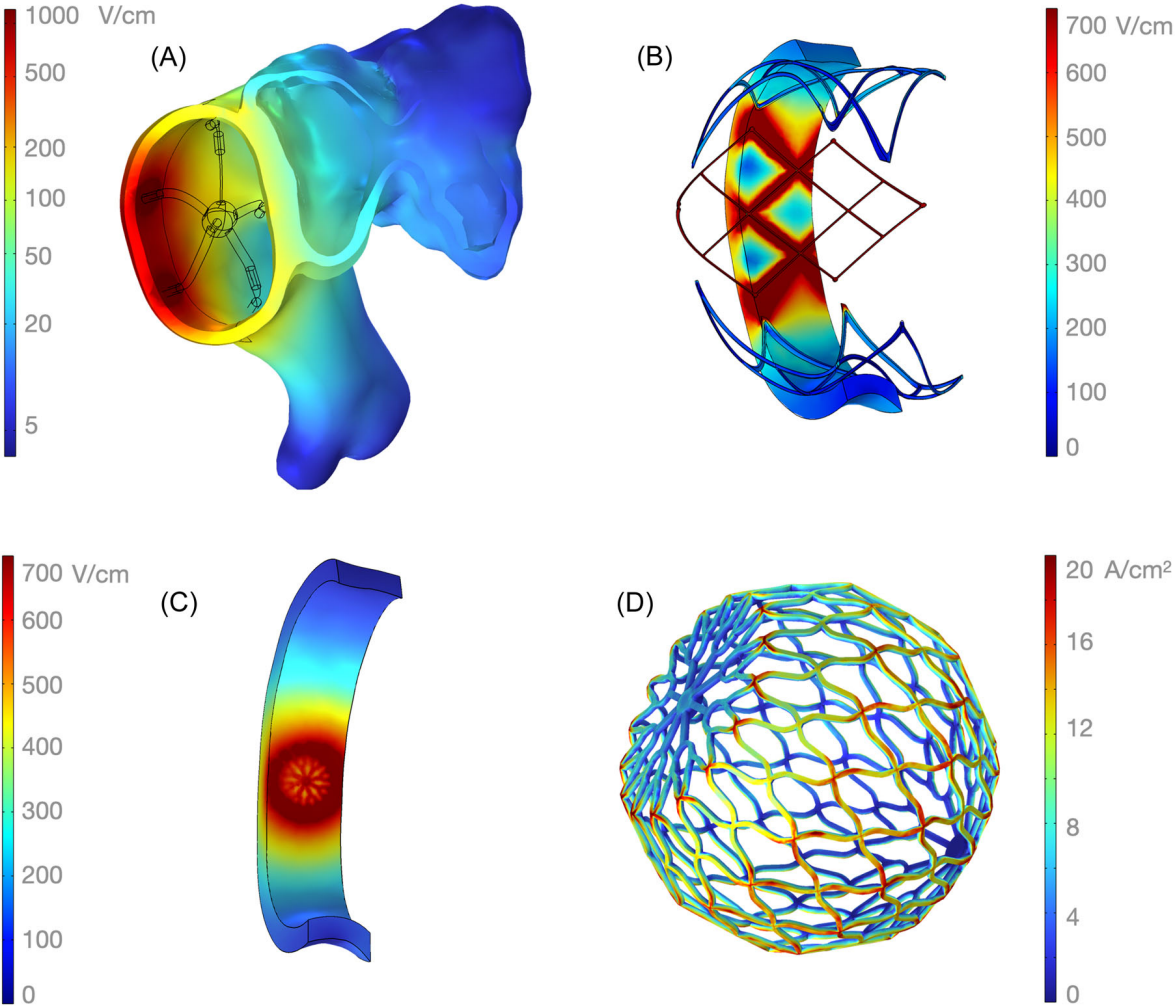
Result samples. (A) the penta‐spline's electric fields at 1800 V, with one of its vectors energized posteriorly, as seen looking into the LPV. LAA is seen anteriorly. (B) Center panel of a large 1‐shot sphere energized at 40 A, on target, showing a “cage effect” that diminishes its efficacy. Tissue inside a wire “diamond” has equal voltages around it, a situation that does not favor current flow in its center. (C) The 9 mm sphere (here at 20 A) has the same type of weakness, to a lesser degree, in the flat area that is pressed against tissue. The cage weakness can be overcome using more current. (D) 9 mm sphere in detail, (20 A), showing current density on the mesh electrode. Red indicates higher current flows at sharp corners. The interior of the sphere and planar tissue‐apposition region have less current.

For comparison purposes, and to control the variable of contact and force equally across catheters, we positioned all of them ideally, in perfect tangential contact with the tissues, centered over the same target, see Figure 2. No catheter electrode deformed the tissue, and none was separated from it either. The 9 mm sphere was planar in part, see Figure 4.

### 2.6 Safety and efficiency outcomes

In therapies that depend on energy delivery, such as PFA and RF ablation, it is easy to see that in the extreme case of an enormous amount of energy delivered, there is a likelihood of very high efficacy, but low safety given the consequent collateral damage. We therefore evaluated three metrics.

First, the dosage required to achieve 90% transmurality is an assessment aimed to elucidate which catheter designs accomplish the objective with less energy input, and is thus a metric of safety. To be precise, we measured the electric current at which 90% of the target's volume had an electric field of more than 600 V/cm, which is a conservative electroporation threshold (upper bound of lethal myocardial cell threshold found in human hearts, see Figure 6A of ref. ^19^).

Second, to compare the potential for bubble formation and excessive heat we obtained the electrode current density (ECD) of the catheters, when equally energized for 90% transmurality. This ECD was defined locally as the current of an individual vector divided by its electrode area, with units A/cm^2^. For the monopolar Nitinol 9 mm and large 1‐shot catheters, the metal areas of the whole cage and a diamond panel, respectively, were used. For the penta‐spline, the total electrode area of a single spline was used. For the rest of the catheters (not monopolar), the area of a single electrode was used. Areas were estimated from publications or manuals (see Table 1). Any gas formation is related to ECD via electrochemical laws. Heat is related to joule heating in Watts/cc (product of the local current density squared and local resistivity). Naturally, a large current concentrated in a very small region will be a heat source.

**Table 1:** Electrode current density with equal lesion formation.^a^

	Current for 90% transmurality	Electrode area	ECD current density	N vectors to cover target (see Figure 2)	ECD x N
	Amps	cm^2	A/cm^2		
Large 1‐shot sphere	70.0	0.57	124	3	371
9 mm sphere^b^	39.0	1.55	25	7	176
Penta‐spline basket	36.1	0.49	74	6	442
Circular	12.5	0.15	83	3	249
Flex splines	5.3	0.13	41	8	324
Balloon	4.0	0.13	31	8	247

a Expresses risk of excessive local heat, bubbles.

b Area excludes inner cage surfaces, which do not source current due to Faraday cage effect, see Figure 3.

Third, we measured the percentage of generator power that goes into the target, an intuitive efficiency metric denoting how much energy is focused on the target, and how much is delivered to nontarget blood and tissue. To be precise, power in target is calculated as the integral of the square of current density (in Amps/square cm) through it multiplied by its tissue resistivity (in ohms cm), a conventional power dissipation metric. The total power delivered is simply the product of current and voltage that the generator injects into the body. Taking the ratio of these two yields the cited efficiency percentage.

Clearly, a catheter that is able to focus its delivery predominantly on the target will be more efficient and have less extra‐atrial effects, such as bubble formation, coronary spasms, phrenic nerve effects, muscle stimulation, hemolysis, and so forth.

## 3 RESULTS

Catheter designs varied markedly in the lesions they produce. Figure 4 shows some examples. A striking result was the “cage” effect of the diamond panels of the large 1‐shot sphere catheter. As is the case with Faraday cages, tissue in the middle of a surrounding diamond will not be exposed to a voltage differential, tissue there is “guarded,” so that no significant current will flow through it. This flow is required to set up the electroporating field that yields lesions. The cage effect can be overcome by substantial increases in energy, but the effect remains a gross inefficiency factor, as the results below will show. The 9 mm focal sphere shows a similar phenomenon, to a lesser degree from a smaller size of openings in the nitinol mesh. It can be compared to placing a coin as an electrode against tissue. Current will be low underneath the coin center, and will be maximal at the perimeter edges.

**Figure 5:**
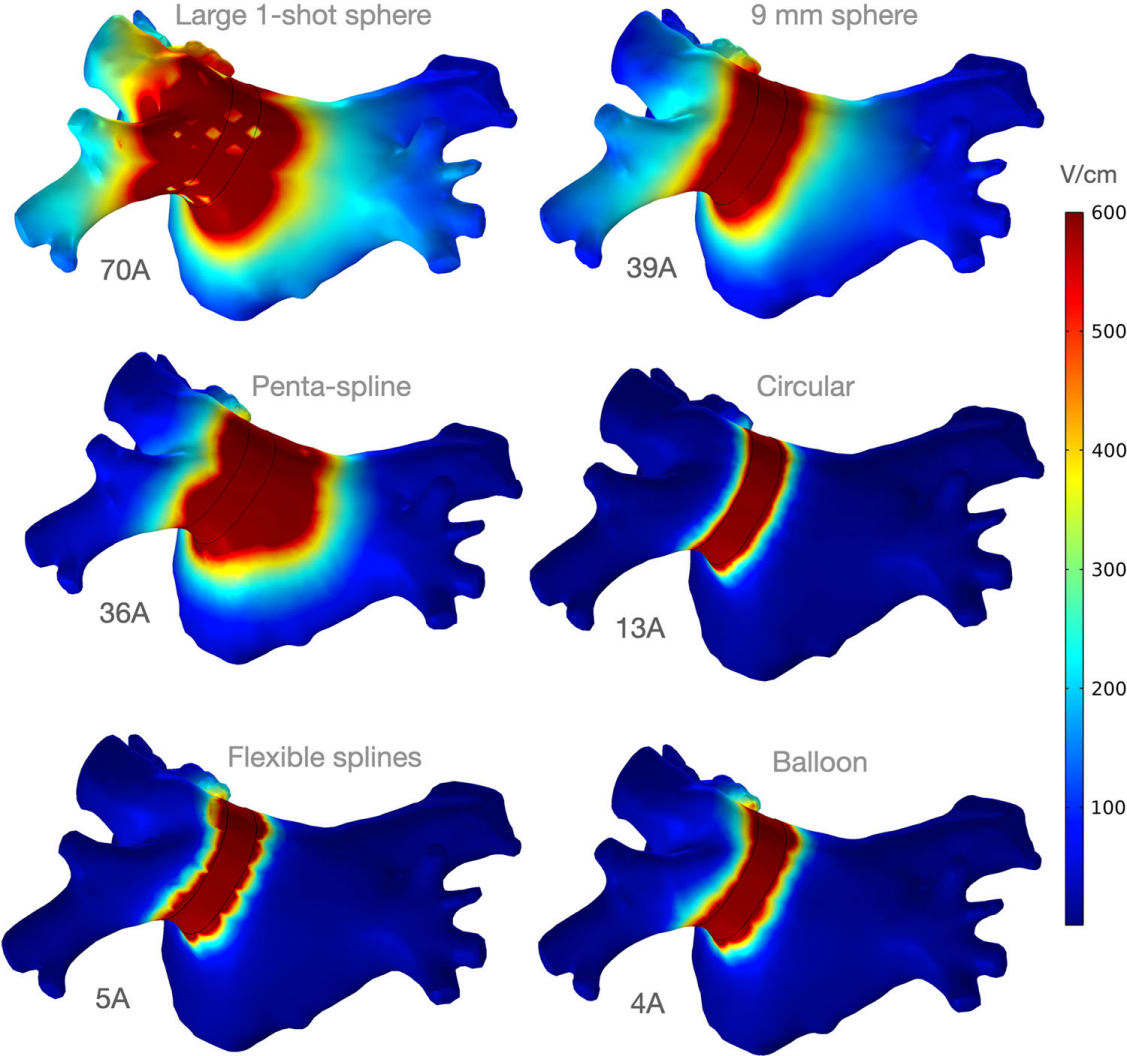
Posterior LA view of lesions for the catheters compared. They are all equally energized to achieve 90% transmurality in the posterior target of the LPV at the current noted (A, Amperes per vector). The less efficient catheters have larger lesions, with more blood shunting, and accordingly require greater current to achieve transmurality.

Addressing one of our two primary outcomes (current for transmurality), Figure 5 shows the lesions created by the six catheters being compared, at equal currents to achieve 90% transmurality in the selected target. It is easy to discern which designs are less efficient and which better focus the generator's energy on the target. Designs such as the large 1‐shot sphere and the penta‐spline tend to ablate a large total area of tissue to achieve PVI, while designs such as the circular catheter, the flexible splines catheter and the balloon tend to ablate a relatively discrete region of tissue at the pulmonary vein (PV) antrum.

**Figure 6:**
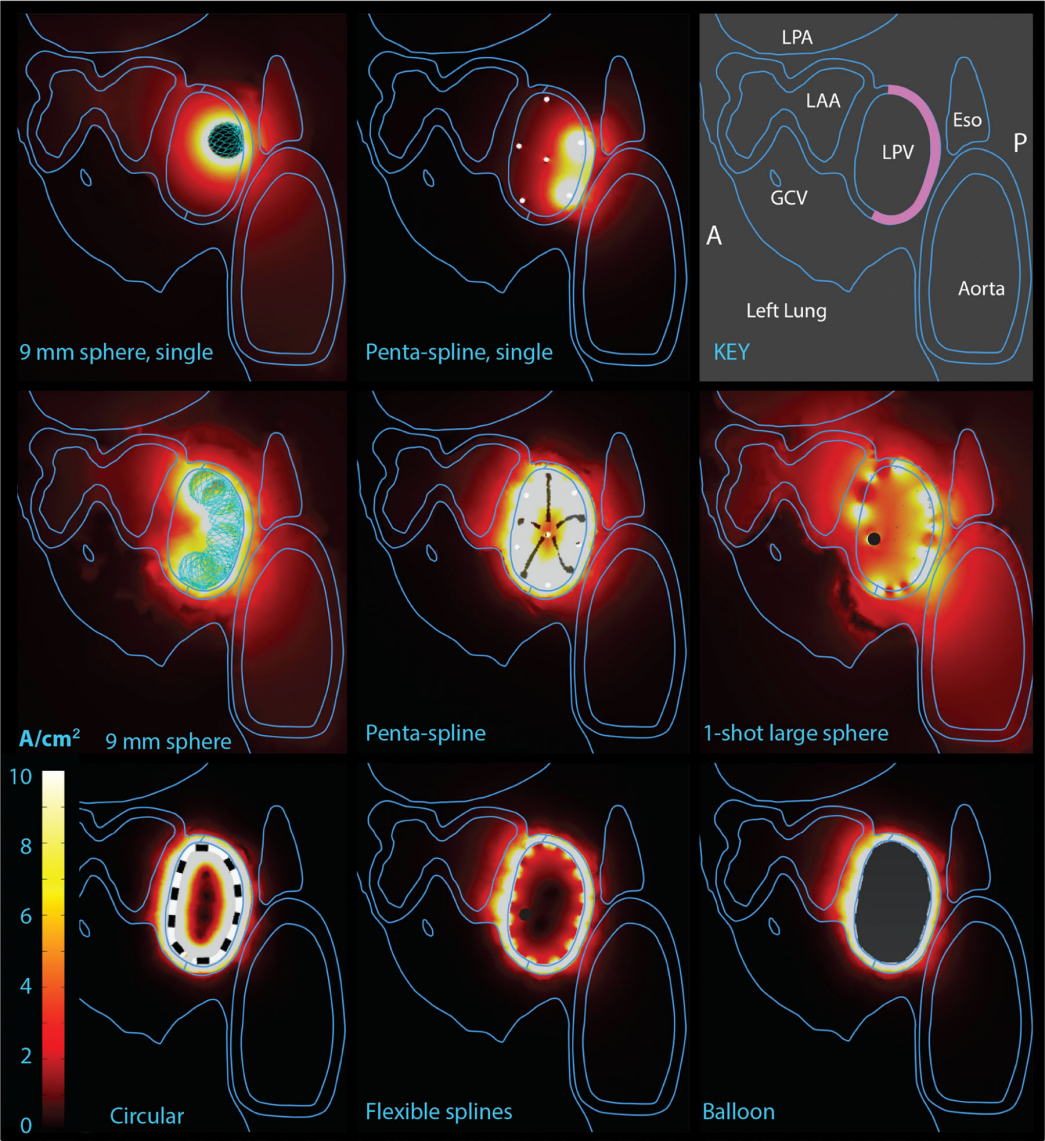
Current flow in blood. Current at the energy for 90% transmurality, in an oblique plane bisecting the target and electrodes in the LPV (per key, top right. Target in purple). All plotted at same scale. Ideally, current should be confined to the atrial wall and avoid blood and nontarget tissues. The 9 mm sphere and penta‐spline are shown during a single vector delivery (top row), and in the middle row with their multiple vectors, as required to cover the length of the target. Consistent with Figure 5, the less efficient catheters have current flow through blood and extra‐atrial tissues. A, anterior; Eso, esophagus; GCV, great cardiac vein; LPA, left pulmonary artery; LAA, left atrial appendage; LPV, left pulmonary vein; P, posterior.

To illustrate where the current is flowing in the different designs, Figure 6 shows current flow in an oblique plane intersecting the target and the electrodes, again energized equally to achieve 90% transmurality. The most efficient catheters keep the current confined to the atrial wall, away from blood, while the less efficient ones have an indiscriminate energy delivery, more likely to affect neighboring tissues and blood. The circular catheter, with its negative and positive electrodes close together, has high shunting currents in blood.

**Figure 7:**
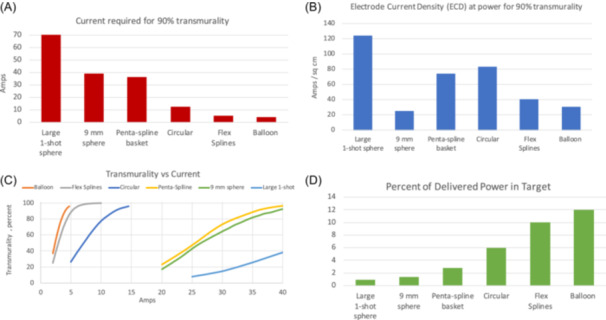
Main results. The top left chart (A) shows the current (per vector) required to achieve transmurality. Less current indicates greater safety, as collateral effects are less likely. The curves (bottom left, C) show a large range of ease with which catheters reach transmurality. The top right chart (B) shows the electrode current density (ECD), allowing a relative comparison of risk for excessive heat and bubbles. Catheters with large area electrodes or high efficiency have less ECD. The bottom right chart (D) shows the efficiency of the catheters.

Figure 7 summarizes the main results quantitatively. The variation across catheters in safety and efficiency, (understood as the percent power in target, or the amount of current required to achieve the lesion), is remarkable.

For readers interested in numerical details of the simulations, the raw data resulting from them can be found in Supporting Information S1: Supplement 1.

## 4 DISCUSSION

### 4.1 Consistency of results with clinical practice

Our summary charts in Figure 7 show a wide variation of efficiency among the catheters compared. Since it is known that PFA has strength‐duration efficacy characteristics,^20^ and that repetition of dose increases the lesion size,^21^ it can be expected that less efficient designs will in practice use lengthier pulse waveforms and trains, higher energy application, or require more dose repetitions. For catheters for which there is enough such information published, for example, electrogram tracings in live conference cases, we compiled energy durations in Table 2 (see Supporting Information S1: Supplement 2 for sources). Other sources informing it were the Advent and Pulsed AF pivotal study publications.^22^, ^23^ Table 2 shows that indeed, the less efficient catheters (per our study) use longer energy delivery durations.

**Table 2:** Energy delivery duration per PV, per pivotal study protocol or live cases.

		Large 1‐shot	9 mm sphere	Penta‐spline	Circular
	Paced or gated	asynchronous	paced 180 bpm	paced 120 bpm	R‐wave gated
A	Number of Applications, per PV	4	7^a^	8	12
B	Number of deliveries per application	30	12	5	4
C	Delivery Duration, msecs	150	150	200	130
ABC	Total delivery duration, msecs	18 000	12 600	8000	6240

a 14 focal applications every 6 mm to isolate LPV antrum in the anatomy presented. Half (7) corresponds to one PV.

### 4.2 Comparison with animal in‐vivo data

As with any modeling study, it is important to validate modeled results with in‐vivo data. Published animal studies using several of the catheters we modeled support our findings regarding lesion dimensions that we demonstrate in Figure 5. Specifically, animal data from the large 1‐shot sphere demonstrate very large lesions encompassing much of the posterior left atrium in addition to the pulmonary veins.^7^ Data from the penta‐spline catheter ^24^ and 9 mm sphere ^3^ demonstrate intermediate‐sized lesions. Studies with two different circular catheters ^25^, ^26^ demonstrate the most discrete lesions. These results are all consistent with our modeled results. A future dedicated in‐vivo study of these catheters comparing safety measures such as hemolysis, damage to surrounding structures, and silent cerebral events in addition to lesions dimensions would be useful to evaluate our modeled results in regard to catheter safety.

### 4.3 Safety and efficiency among catheters studied

As seen in Figure 7, with respect to the dosage required to achieve 90% transmurality, our safety metric, there was a factor of 18X among the catheters studied. Regarding the percentage of generator power that goes into the target, a factor of 13X was found in the comparison.

One would think these two metrics (current for transmurality, percent power in target) convey the same information. However, by varying positioning of the 1‐shot sphere, we observed that the percent power into target could remain largely unchanged, while the current required for transmurality could vastly increase. The cause of this behavior was the “cage effect” holes discussed previously, and their position. As this example illustrates, our two outcome metrics are not just a converse of each other. In clinical use, altering catheter position (by rotating or otherwise moving a catheter) in between energy applications in a given PV or region may help to overcome inefficiencies in catheter design, such as the “cage effect,” by spreading ablative energy to tissue not fully ablated in the initial application. Additionally, the very wide lesions produced by some catheters compared to others (Figure 5) will tend to create redundancy of ablated tissue around a PV which may facilitate achievement of PVI and enhance its durability, at the possible expense of contractile function and a great total volume of ablated myocardium.

### 4.4 Relevance of our results to PFA side effects

Our study provides a comparison of efficiency across catheter designs. Accomplishing efficient ablations with a target‐focused approach is important to minimize undesired effects that are concerning during today's PFA procedures. Coronary spasm, hemoglobinuria, kidney injury, phrenic nerve injuries, coughing, conduction blocks, pain, and skeletal muscle stimulation are all problems that necessarily increase with dose (a zero dose has none of these, while an enormous dose may have all). Some of these problems are being addressed with stopgap measures such as in‐procedure nitroglycerine, atropine, increased levels of anesthesia and paralytics, and increased hydration.

Embolizing microbubbles will also increase with energy dose, for any catheter, as mandated by electrochemical laws. Catheters with large areas (Table 1), or those using less current to achieve a lesion, are necessarily smaller bubble producers, as shown in Figure 7. The recently published Advent trial reported 9% silent cerebral events or lesions in the PFA arm, compared to zero on the thermal ablation arm.^22^ The relevance of these cerebral lesions is controversial today, with some contending that embolic events are not of clinical importance.^23^ Others argue that there has been inadequate neurological and MRI assessment in PFA clinical studies, with too few imaged patients, or not enough postprocedure wait time for fluid‐attenuated inversion recovery (FLAIR) positivity.^27^, ^28^ The Pulsed AF trial had 15% of patients undergo an MRI, 8% of them had cerebral lesions.^23^ In the Advent trial, 11% of the patients in the PFA arm had an MRI.^22^ In both studies, MRI timing is not well reported, with the majority probably imaged within 24 h per common clinical study logistics. This contrasts with serial MRI data in dogs that showed diffusion weighted imaging (DWI) signals decreasing through Day 4 post gas emboli, while FLAIR signals increased in the same time period.^29^ Neurocognitive assessments are variable in PFA clinical studies, present in some and absent in others.^22^, ^23^ Hemolysis may also be encountered with very high energy PFA deliveries,^4^ and with high ECD at the ablative electrodes which are in contact with blood. Finally, in this partial list of side effects, a randomized study in 65 patients found 10 times more myocardial damage (troponin 10 102 vs 1006 ng/L) in PFA than RF ablation.^5^

### 4.5 Application of computer modeling to clinical scenarios

Computer modeling can provide useful information to predict and interpret clinical effects of electrophysiologic tools. In the case of this current study, we have modeled the delivery of energy by different PFA catheter designs to the left atrial and pulmonary vein target (which is desirable) as well as shunting of energy to surrounding structures (which is undesirable). We have also modeled the energy required for different catheter designs to reach target tissue voltage gradients which have been previously demonstrated to cause irreversible electroporation. We have demonstrated large differences between different catheter designs in these parameters, which ultimately will impact lesions characteristics as well as energy shunting to nontarget tissue which may increase the risk for complications. Pulse waveforms (which remain proprietary within each company and so are not currently available for modeling) will also play a role in target lesion formation and complication risk, but the studies we have performed can accurately model efficiency of each catheter's energy delivery to target tissue vs surrounding structures (given our stated assumptions about catheter orientation and contact), in a manner that is not dependent on energy waveform.

In regard to clinical applicability of computer models, we have previously studied subcutaneous defibrillator efficiency in regard to defibrillation energy application to target tissue (the ventricles) versus surrounding structures.^30^ These modeling results were directly used to create the PRAETORIAN score, which was found to accurately estimate clinical defibrillation efficacy.^31^ We then performed further biophysical modeling of the defibrillation energy efficiency of different subcutaneous defibrillation electrode configurations,^32^ and our modeled results were found to accurately predict defibrillation efficacy in humans.^33^ We believe that the modeling of different PFA electrode configurations we have performed in this manuscript can provide similarly instructive information to understand energy delivery to tissue and thereby clinical behavior of these different PFA catheter designs, even in the absence of proprietary waveform information.

### 4.6 Applicability to RF ablation

Our study has focused on PFA, and modeled lesions as tissue having a certain electric field strength (e.g., 600 V/cm) like other modeling investigations have done.^1^, ^19^, ^34^ No significant thermal mechanisms are expected with PFA and none were modeled here. Radio frequency ablation (RFA) on the other hand does rely on thermal mechanisms, with longer energy applications of lower voltage and current. The modeling of RFA utilizes heat generation as the creator of lesions. A time‐dependent model defines lesions as tissues that reach certain temperatures with time. Our models are quite capable of representing this. We recognize that some of the catheters modeled in our study are dual‐modality catheters, PFA or RFA through the same electrode. However, modeling RFA lesions has been reported before (one of several good examples is reference ^35^), and was beyond the scope of this study.

## 5 STUDY LIMITATIONS

### 5.1 An ideal computer model versus varied clinical practice

Computer modeling studies such as ours are often used in catheter research and development.^1^, ^19^, ^34^ They keep all anatomical, respiratory, operator, tissue thickness, and device placement variables exactly constant and perfectly controlled across catheter comparisons, a virtue that allows a meaningful comparison of an experimental variable (e.g., efficiency) despite an *N* = 1. However, in clinical practice there is a range of anatomies and tissue thickness. Endless variations in nonideal catheter contact with different operators are possible. These variations are not easily amenable to computer modeling and require in‐vivo preclinical and clinical data with large sample size to allow catheter comparisons. Such an in‐vivo study would require active collaborative participation by several device companies, as all but two of the catheter designs that we modeled are currently investigational. Until such difficult and expensive in‐vivo study becomes a reality, computer modeling remains a modest but important step to both direct and interpret in‐vivo experimentation.

### 5.2 Uncertainty on actual waveforms and vectoring

In the context of manufacturer secrecy regarding PFA waveform characteristics, we have designed a modeling study that does not require waveform specifications. Regarding vectoring, which we do need, when no direct publication existed, we estimated vectoring with reasonable confidence based on published information combined with live and prerecorded case transmissions at scientific meetings. When in doubt about PFA vectoring, we modeled several variations (as we did for the 1‐shot sphere and the penta‐spline) and selected the best performer for comparison.

## 6 CONCLUSIONS

We do not venture to make claims about actual in‐vivo clinical results, given the limitations described and the modeling‐only scope of our study. We can only conclude that our computer models suggest a large variation among the compared catheters with respect to lesion size and efficiency when evaluated by the energy required to achieve transmurality, and percent power into target. While our findings are consistent with published animal studies and pivotal clinical study energy durations, further confirmation with in‐vivo data is in order (Central Illustration 1).

**Central Illustration 1:**
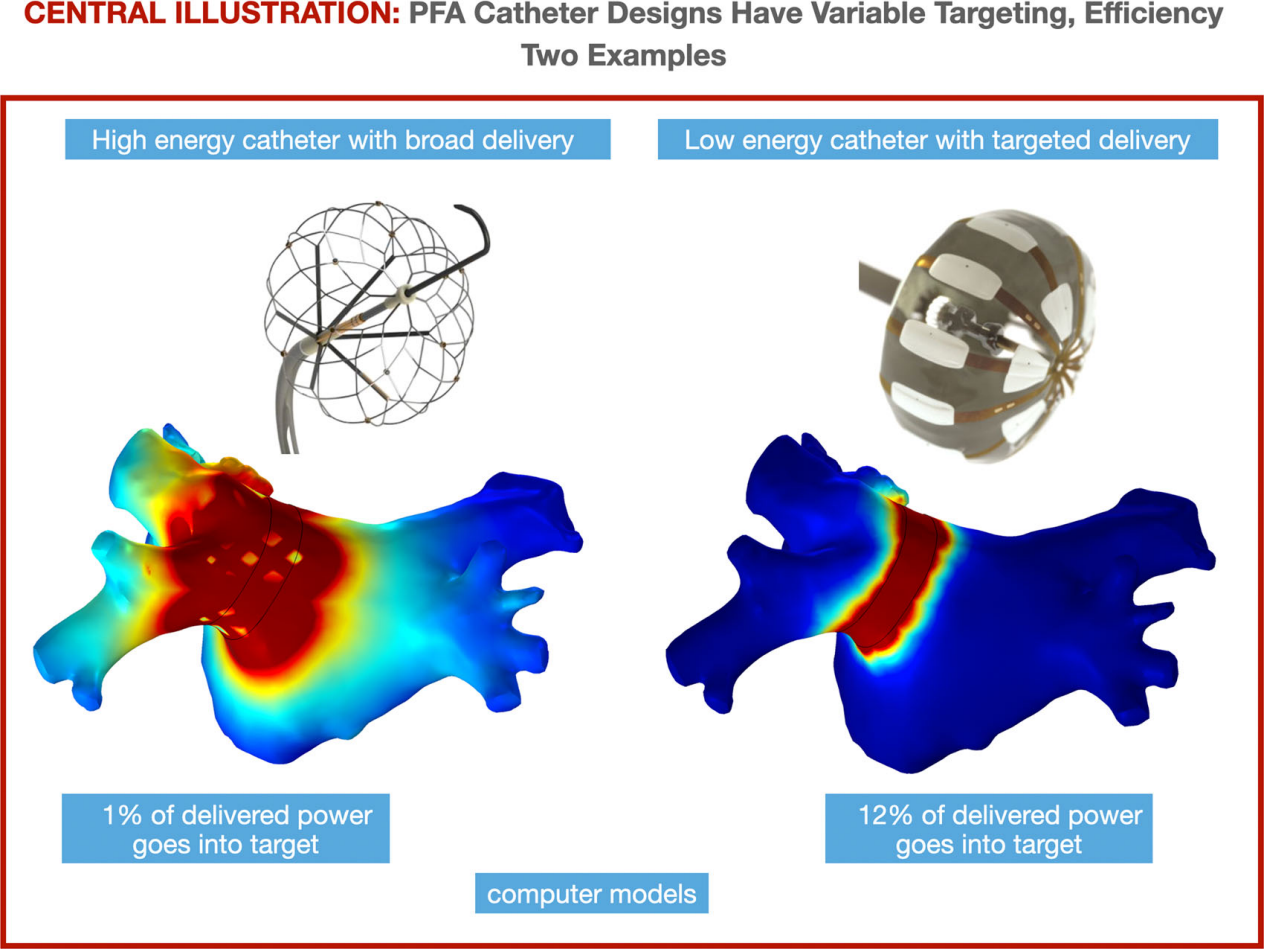
PFA catheter designs have variable targeting and efficiency: two examples.

## Supporting information

Supporting information.

## Data Availability

The data that support the findings of this study are available from the corresponding author upon reasonable request.
