# Supplementary material for: Comparison of efficiency of PFA catheter designs by computer modeling
Source: J Cardiovasc Electrophysiol. 2024 Oct 8;35(12):2382–93. doi: 10.1111/jce.16459 (PMC11650406; doi:10.1111/jce.16459)
Supplement: Supplementary file 1 — Supporting information. [file JCE-35-2382-s001.pdf]

Supplement 1, Raw Simulation Results

Penta-spline efficiency data

2 applications, 1 intervening rotation, 10 sequential pairs, 6 adjacent sequential pairs on target

	Current	Impedance	Total Power	Power in target	percent
Pair	A	Ω	Watts	Watts	
1 to 3	40.6	49.3	81,198	117.3	0.1%
9 to 1	40.6	49.3	81,182	470.1	0.6%
2 to 4	41.04	48.7	82,075	870.5	1.1%
10 to 2	40.46	49.4	80,917	45.6	0.1%
5 to 7	41.84	47.8	83,678	2806.1	3.4%
6 to 8	38.96	51.3	77,913	2709.6	3.5%
3 to 5	41.06	48.7	82,104	1747.0	2.1%
7 to 9	37.9	52.8	75,842	2267.0	3.0%
4 to 6	40.85	49.0	81,701	2541.1	3.1%
8 to 10	40.1	49.9	80,175	1078.4	1.3%
Totals	403.41		806,786	14652.7	1.8%
Average	40.34	49.61	80,679	1465.3	1.8%
Totals for pairs on target:		49.9	481,414	13,149	2.73%

(non-yellow pairs are on anterior LA wall. They do not contribute to target, so are not counted)

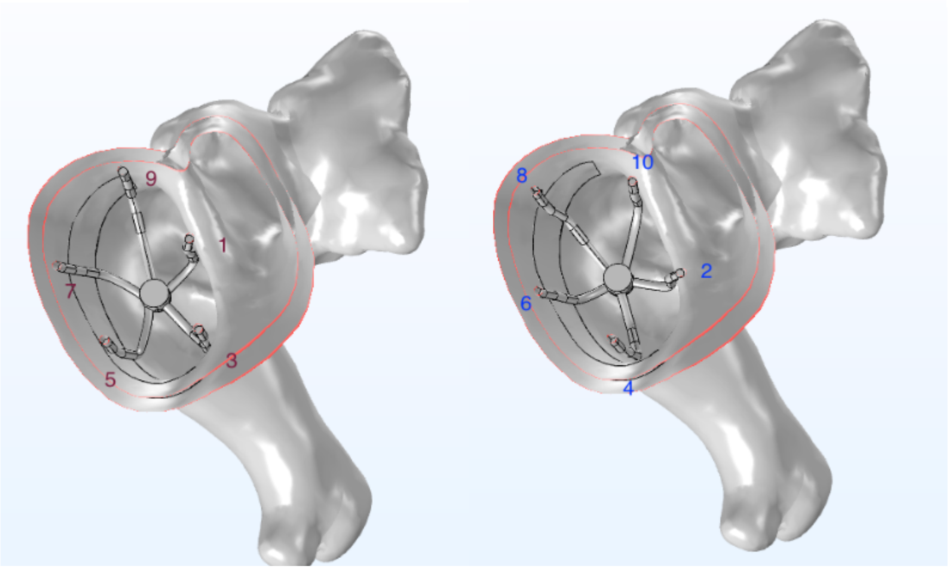

Penta-spline, Transmurality vs Current

Current	Voltage (V)	Treated Vol (mm^3)	Transm.
20.0	1000	131.35	23.08
22.0	1100	183.92	32.32
24.0	1200	239.31	42.05
26.0	1300	299.46	52.62
28.1	1400	362.95	63.78
30.1	1500	417.15	73.30
32.1	1600	456.02	80.13
34.1	1700	488.03	85.76
36.1	1800	517.7	90.97
38.1	1900	535.8	94.15
40.1	2000	547.73	96.25

Average impedance:	49.9	ohms
Current for 90% transmurality:	36.1	Amps
Electrode metal area, 1 spline:	0.49008845	cm^2
ECD:	73.5910445	A/cm^2

9 mm sphere, efficiency data

position	Total current A	Volts	Impedance Ω	Power Watts	in target Watts	
S3	39	2132.4	54.678	83,165	818.38	
S2	39	2103.7	53.942	82,046	1102.7	
S1	39	2060.3	52.827	80,350	1278	
M	39	2034.9	52.178	79,363	1298	
L1	39	2054.2	52.673	80,116	1302.7	
L2	39	2093.9	53.69	81,662	1148.5	
L3	39	2114.9	54.229	82,482	827.01	
	Ave V:	2085	Totals:	569,184	7,775	1.37%

7 positions to cover target, given every 6 mm:

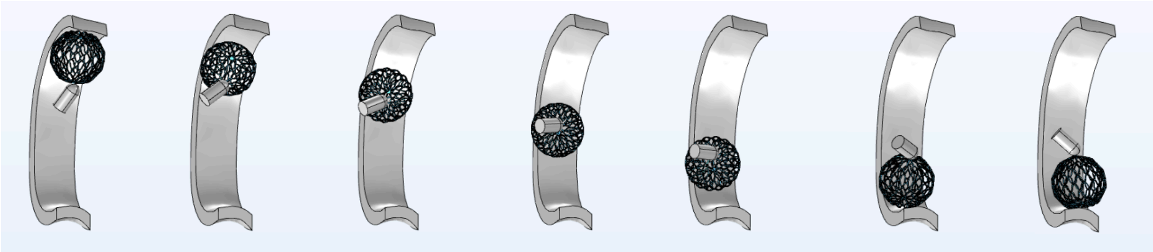

S3 S2 S1 M L1 L2 L3

9 mm sphere, transmurality vs current

Current (A)	Treated (mm^3)	Transm. (%)
20	98.473	17.2817
23	181.18	31.7966
26	273.44	47.9879
30	367.49	64.4934
31	388.25	68.1367
32	411.84	72.2767
33	429.44	75.3655
34	448.8	78.7631
35	466.09	81.7974
36	479.26	84.1087
37	494.19	86.7289
38	501.76	88.0574
39	514.59	90.3091
40	525.84	92.2834
42	540.94	94.9334
44	550.69	96.6445
46	558.89	98.0836
48	562.01	98.6311
50	564.51	99.0699

current for 90% transmurality:	39	A
Electrode metal surface area*:	1.55	cm^2
ECD:	25.16	A/cm^2

* Excludes inner surfaces, they don't source appreciable current due to Faraday cage effect. See Figure 3D in manuscript.

Circular catheter, efficiency data

Source and sink electrodes are interlaced

Current at source (+) electrodes:	12.5	A
x 3 source electrodes against target:	37.5	A
Electrode Impedance, average:	113.3	ohms
Voltage, average:	1417	volts
Total power delivered by 3 source elecs.:	159,370	watts
Power into target at 12.5 A:	9,422.6	watts
Efficiency, power in target/total power:	5.91%	

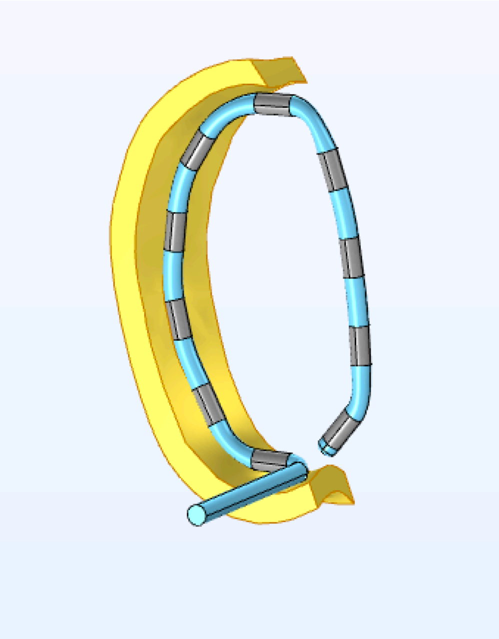

Circular catheter, Transmurality vs Current

Current (A)	Treated (mm^3)	Transm. (%)
5.0	153.59	26.749
5.5	186.43	32.468
6.0	219.44	38.217
6.5	250.16	43.567
7.0	280.97	48.933
7.5	311.19	54.196
8.0	342.34	59.621
8.5	370.52	64.529
9.0	397.7	69.263
9.5	422.79	73.632
10.0	445.09	77.516
10.5	464	80.809
11.0	479.55	83.518
11.5	495.19	86.241
12.0	509.28	88.695
12.5	521.05	90.745
13.0	530.89	92.459
13.5	537.56	93.621
14.0	543.21	94.605
14.5	548.75	95.569
15.0	553.26	96.355
15.5	557.13	97.029
16.0	563.05	98.060
16.5	564.77	98.359
17.0	566.28	98.622
17.5	567.29	98.798
18.0	567.99	98.920
18.5	568.95	99.087
19.0	569.88	99.249
19.5	571.15	99.471
20.0	571.94	99.608

Current per source electrode at 90% transmurality:	12.5	A
Area, 1 electrode of 1.6 mm dia, 3 mm length:	0.1508	cm^2
ECD:	82.89	A/cm^2

Flex Spline Catheter, Efficiency data:
2 applications, electronic rotation, interlaced source & sink elecs.
with inactive elecs between sources and sinks

Current at source electrodes at 90% transm:	5.25	A
x 4 source electrodes against target:	21	A
Electrode Impedance, average:	229.03	ohms
Voltage at source electrodes, average:	1202.4	volts
Power delivered:	101,002	watts
Power into target at 5.25 A:	10,093	watts
Efficiency, power in target/total power:	9.99%	

Current per source electrode for 90% transm:	5.25	A
Area, electrode of 3.6x3.6 mm:	0.1296	cm^2
ECD:	40.51	A/cm^2

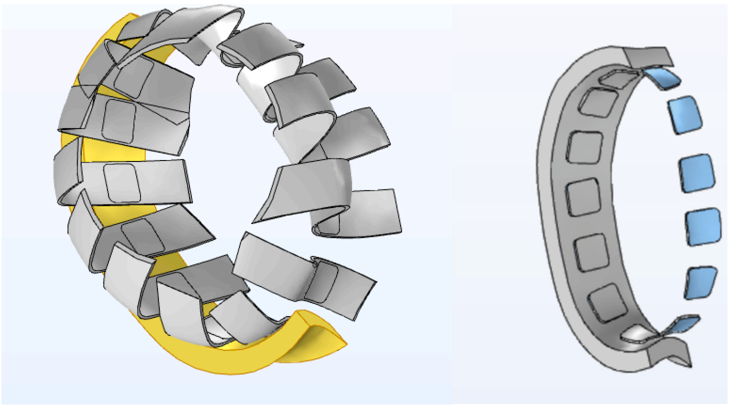

With a wide interlaced delivery there are 2 sources on the target in each of the two applications.
(every fourth electrode is a source, and every fourth electrode is a sink)
total of 4 sources on the target. See ref (4).

Flex Spline Catheter, Transmurality vs current data:

Current (A)	Treated Vol (mm^3)	Transm. (%)
2	143.12	25.136
2.25	183.38	32.208
2.5	223.54	39.261
2.75	259.25	45.533
3	296.97	52.158
3.25	330.83	58.105
3.5	365.48	64.191
3.75	396.36	69.615
4	422.78	74.255
4.25	446.79	78.472
4.5	469.43	82.448
4.75	489.09	85.902
5	503.6	88.449
5.25	516.78	90.765
5.5	527.78	92.697
5.75	535.53	94.058
6	542.32	95.25
6.25	548.35	96.31
6.5	552.1	96.968
6.75	555.83	97.624
7	558.26	98.051
7.25	560.41	98.428
7.5	562.19	98.742
7.75	563.6	98.988
8	564.76	99.192
8.25	565.74	99.363
8.5	566.53	99.503
8.75	567.15	99.612
9	567.54	99.68
9.25	567.9	99.743
9.5	568.1	99.779
9.75	568.58	99.863
10	568.7	99.884

Balloon Catheter, Efficiency data:
2 applications, electronic rotation, interlaced source & sink elecs
with inactive elecs between sources and sinks

Current at source electrodes at 90% transm:	4	A
x 4 source electrodes against target:	16	A
Electrode Impedance, average:	280.26	ohms
Voltage at source electrodes, average:	1,121	volts
Power delivered:	71,745	watts
Power into target at 4 A:	8,603	watts
Efficiency, power in target/total power:	11.99%	

Current per electrode for 90% transm:	4	A
Area, 1 electrode of 3.6x3.6 mm:	0.1296	cm^2
ECD:	30.86	A/cm^2

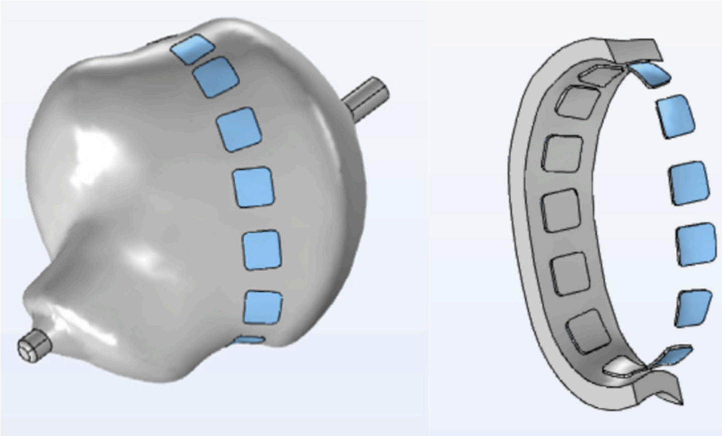

With a wide interlaced delivery there are 2 sources on the target in each of the two applications.
(every fourth electrode is a source, and every fourth electrode is a sink)
total of 4 sources on the target

Balloon Catheter, Transmurality vs current data:

Current (A)	Treated Vol (mm^3)	Transm. (%)
2	211.83	37.208
2.25	259.48	45.576
2.5	308.82	54.243
2.75	352.34	61.886
3	393.64	69.142
3.25	429.99	75.525
3.5	459.78	80.758
3.75	486.05	85.372
4	507.13	89.075
4.25	524.13	92.06
4.5	536.3	94.198
4.75	544.72	95.678
5	550.57	96.706
5.25	556.64	97.77
5.5	559.79	98.324
5.75	562.66	98.828
6	564.4	99.135
6.25	565.94	99.405
6.5	566.97	99.585
6.75	567.65	99.704
7	568.15	99.794
7.25	568.59	99.871
7.5	568.79	99.906
7.75	568.98	99.938
8	569.08	99.956
8.25	569.17	99.972
8.5	569.2	99.977
8.75	569.2	99.977
9	569.21	99.979
9.25	569.24	99.984
9.5	569.24	99.984
9.75	569.24	99.984
10	569.24	99.984

Large Nitinol sphere, single shot: efficiency data
sequential unipolar, 3 deliveries

Panel	Total Current A	Impedance Ω	Total Power Watts	Power into target Watts	percent
P1	70	47.1	230,937	2,797	1.2%
P2	70	46.7	228,634	1,080	0.5%
P3	70	47.0	230,300	2,133	0.9%
Totals			689,871	6,010	0.9%

Current for 90% transmurality:	70.0	Amps
Area, metal, 1 panel	0.5655	cm^2
ECD:	123.78	A/cm^2

3 panels cover the target, as seen in Figure 3 B of manuscript. Shown is P1 mid-height, P2 is superior, and P3 inferior:

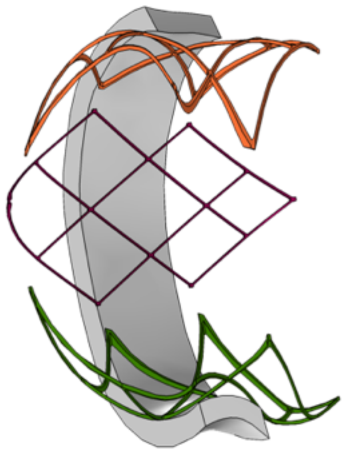

Large Nitinol sphere, Transmurality vs current data:

Current (A)	Treated Vol (mm^3)	Transm. (%)
25	44.178	7.76
30	83.264	14.63
35	145.54	25.56
40	216.63	38.05
45	276.99	48.65
50	333.59	58.59
60	432.66	76.00
70	502.45	88.25
80	540.04	94.86
90	557.34	97.90
100	563.49	98.98

Supplement 2. Energy delivery duration, sources

The following 4 figures illustrate how energy delivery durations were obtained from live-case publications to inform the data presented in Table 1.

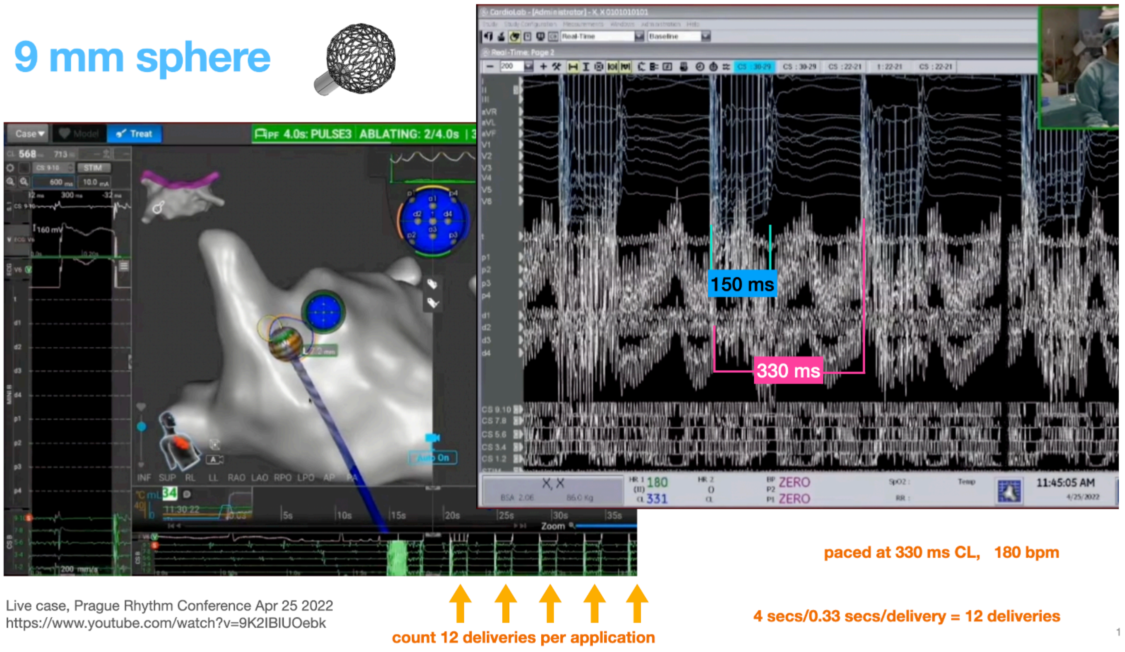

https://www.youtube.com/watch?v=9K2IBlUOebk

Figure Supplement 2.1

Several live cases of the 9 mm nitinol sphere have been shown in the last few years, here is one of them, published at the Youtube link at the lower left. We like this one because it has tracings at 200 mm/sec, good timing resolution. There are 12 deliveries per application. They are timed by pacing at 100 bpm (or 330 msecs cycle length), taking a total 4 seconds per application. From the artifacts, one can see that each PFA delivery has a duration of 150 msecs.

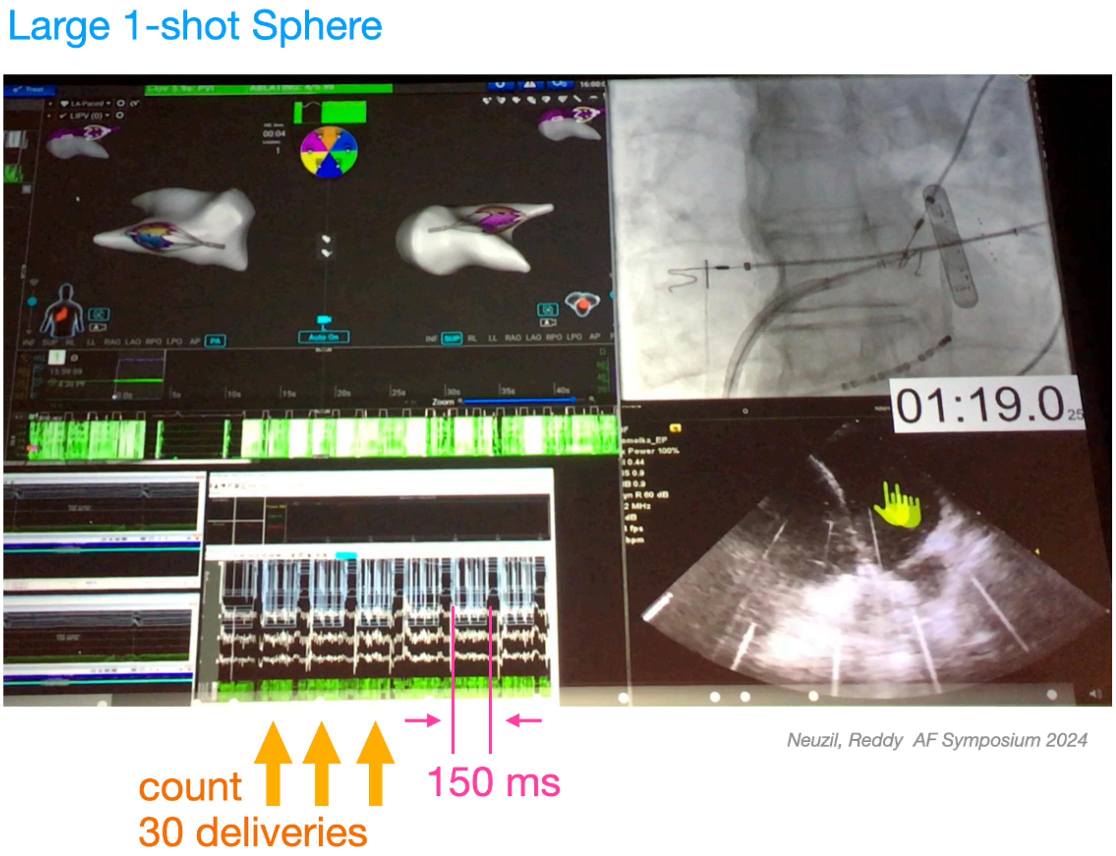

Figure Supplement 2.2

A live case with the Large-1shot device was presented at the Boston AF Symposium 2024 by P Neuzil and V Reddy. Careful observation of the Prucka tracings, aided by the real time clock (with millisecond scale) included in the presentation allowed confirmation that there were 30 energy deliveries, each of 150 milliseconds duration, with each application. 4 applications per pulmonary vein were shown.

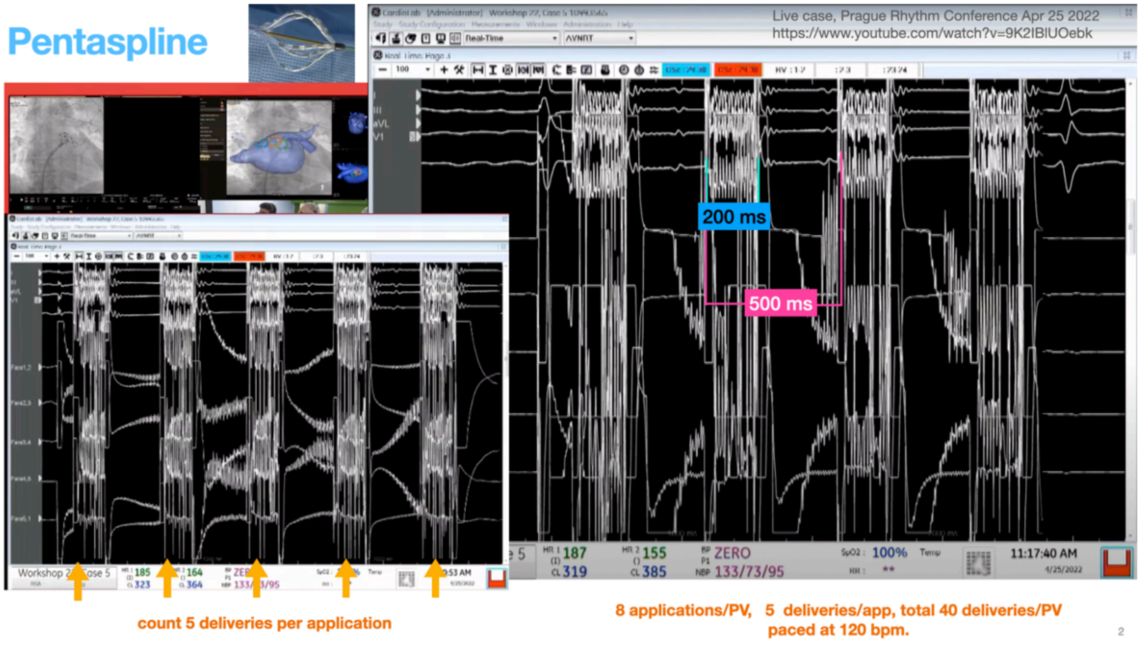

https://www.youtube.com/watch?v=9K2IBlUOebk

Figure Supplement 2.3

The penta-spline device uses 5 deliveries per application, they last about 200 msecs. The clinical study protocol has 8 applications per pulmonary vein, resulting in 40 deliveries per PV.

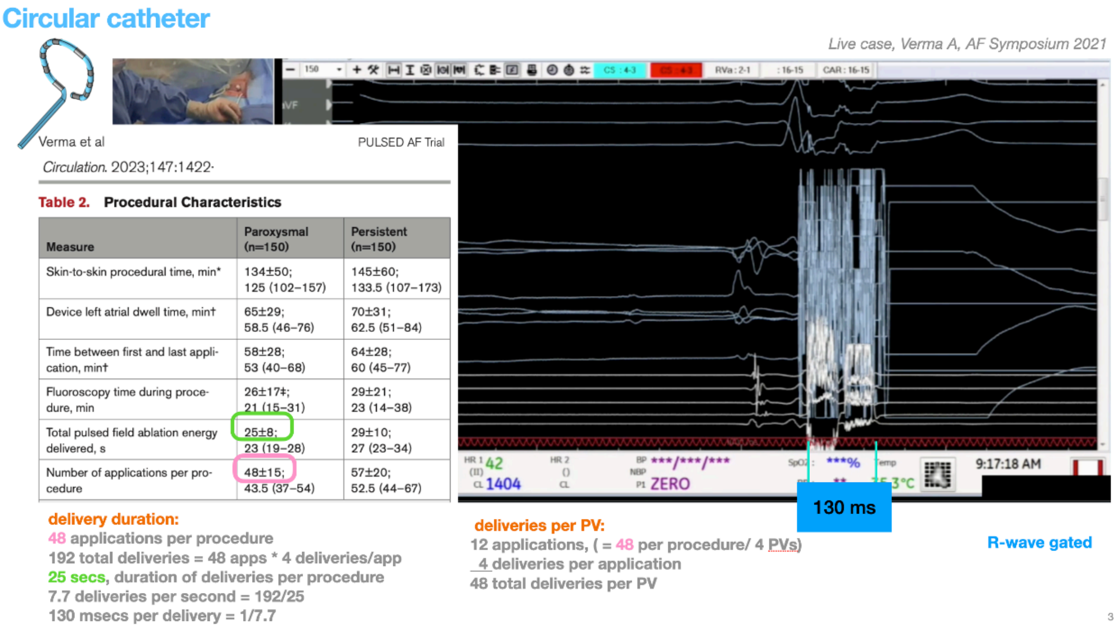

Table 2. Procedural Characteristics

Measure	Paroxysmal (n=150)	Persistent (n=150)
Skin-to-skin procedural time, min*	134±50; 125 (102–157)	145±60; 133.5 (107–173)
Device left atrial dwell time, min†	65±29; 58.5 (46–76)	70±31; 62.5 (51–84)
Time between first and last application, min†	58±28; 53 (40–68)	64±28; 60 (45–77)
Fluoroscopy time during procedure, min	26±17‡; 21 (15–31)	29±21; 23 (14–38)
Total pulsed field ablation energy delivered, s	25±8; 23 (19–28)	29±10; 27 (23–34)
Number of applications per procedure	48±15; 43.5 (37–54)	57±20; 52.5 (44–67)

Figure Supplement 2.4

The manufacturer of this circular catheter discloses more about its energy delivery than others. Vectoring is fully disclosed. The Pulsed AF Trial was recently published and shows these numbers. From a live case Dr Verma presented at the 2021 AF symposium, we measured an energy delivery duration of about 130 msec, which is verified with the numbers on the table, as shown in the above calculations. There were 4 deliveries per application (not shown in figure above, but can be seen in the video). A total of 48 deliveries were given to each PV.
